# Cost-effectiveness of diagnostic imaging modalities in symptomatic patients with lower limb peripheral arterial disease: discrete event simulation model

**DOI:** 10.3389/fpubh.2024.1367447

**Published:** 2024-09-03

**Authors:** Vojtěch Kamenský, Vladimír Rogalewicz, Ondřej Gajdoš, Gleb Donin, Barbora Mašková, Martina Holá, Aleš Tichopád

**Affiliations:** Department of Biomedical Technology, Faculty of Biomedical Engineering, Czech Technical University in Prague, Prague, Czechia

**Keywords:** discrete event simulation, cost-effectiveness, diagnostic modalities, symptomatic lower limb peripheral disease, lifetime costs and effects

## Abstract

**Objective:**

Lower limb peripheral arterial disease in the symptomatic stage has a significant effect on patients´ functional disability. Before an intervention, an imaging diagnostic examination is necessary to determine the extent of the disability. This study evaluates cost-effectiveness of duplex ultrasonography (DUS), digital subtraction angiography (DSA), computed tomography angiography (CTA) and magnetic resonance angiography (MRA) in the diagnostics of symptomatic patients with lower limb peripheral arterial disease indicated for endovascular or surgical intervention.

**Methods:**

Discrete event simulation was used to capture lifetime costs and effects. Costs were calculated from the perspective of the health care payer, and the effects were calculated as quality-adjusted life year’s (QALY’s). The cost-effectiveness analysis was performed to pairwise compare CTA, MRA and DSA with DUS as the baseline diagnostic modality. A scenario analysis and probabilistic sensitivity analysis were carried out to evaluate the robustness of the results.

**Results:**

In the basic case, the DUS diagnostic was the least expensive modality, at a cost of EUR 10,778, compared with EUR 10,804 for CTA, EUR 11,184 for MRA, and EUR 11,460 for DSA. The effects of DUS were estimated at 5.542 QALYs compared with 5.554 QALYs for both CTA and MRA, and 5.562 QALYs for DSA. The final incremental cost-effectiveness ratio (ICER) value of all evaluated modalities was below the cost-effectiveness threshold whereas CTA has the lowest ICER of EUR 2,167 per QALY. However, the results were associated with a large degree of uncertainty, because iterations were spread across all cost-effectiveness quadrants in the probabilistic sensitivity analysis.

**Conclusion:**

For imaging diagnosis of symptomatic patients with lower limb peripheral arterial disease, CTA examination appears to be the most cost-effective strategy with the best ICER value. Baseline diagnostics of the DUS modality has the lowest costs, but also the lowest effects. DSA achieves the highest QALYs, but it is associated with the highest costs.

## 1 Introduction

The study evaluates atherosclerotic disease of lower limb arteries, which can cause partial or complete obstruction of the peripheral artery (1). In developed countries, it is estimated that the prevalence of the peripheral arterial disease (PAD) is between 3 and 10% in the population over 50 years of age, while it rises to 15–20% in the population over 70 years (2).

The disease can manifest with a variety of clinical symptoms ranging from asymptomatic patients to intermittent claudication (IC), or chronic limb-threatening ischemia (CLTI) with possible ulcerations and gangrene (3). Most patients with confirmed lower limb PAD do not have classic symptoms like claudication, but other limb symptoms, or are asymptomatic (4, 5). However, symptomatic patients have significant functional impairment. Claudication is defined as pain in the calf area (it can also appear in the thigh or buttocks) when walking, which does not stop during walking and does not appear when the patient sits or stands. The pain thus forces patients to slow down or stop walking (4, 6).

According to the Inter-Society Consensus for the Management of Peripheral Arterial Disease (TASC II) (2), the prevalence of IC is around 6% in the population of 60-year-old patients. Focusing on the occurrence of IC in patients with low ankle-brachial index (ABI) values, Fowkes et al. (3) mention the results of The Copenhagen City Heart Study, where they found a prevalence of 31% in the 65–74 age group. The authors further state that the prevalence of IC decreases in older age groups, which is probably caused by less mobility of these patients. The prevalence of IC is higher in men than in women.

Patients with IC are limited in physical activities (especially walking), and the goal of their therapy is therefore to provide relief from the pain symptoms associated with IC. The primary treatment approach is structured exercise therapy and pharmacotherapy to modify risk factors and reduce the risk of cardiovascular morbidity and mortality (2). If the patient does not respond to the exercise therapy, revascularization treatment is indicated for patients based on information from imaging methods (2, 4, 6).

Commonly used imaging methods include duplex ultrasonography (DUS), digital subtraction angiography (DSA), computed tomography angiography (CTA), and magnetic resonance angiography (MRA). The choice of the diagnostic modality should be based on patient’s characteristics and the expected size and nature of the impairment. Methods such as CTA and MRA can provide a 3D image of the examined area, whereas DSA only provides a 2D image. Similarly, DUS has limited imaging options, but it is an accessible examination without the use of ionizing radiation and at low cost (7).

There are different diagnostic modalities with different advantages and disadvantages and with different costs associated with the examination. The objective of this study is to evaluate the cost-effectiveness of DUS, DSA, CTA and MRA in the diagnosis of symptomatic patients with lower limb peripheral arterial disease indicated for endovascular or surgical intervention.

## 2 Materials and methods

Previously published discrete event simulation model (8) [used to evaluate cost effectiveness of lower limb PAD screening with measurement of ankle-brachial index (ABI) in asymptomatic patients] was used to evaluate different diagnostic modalities in symptomatic patients. The simulation was carried out during the first half of the year 2022. The model was created using the R programming language in the RStudio software environment (9).

**Figure 1:**
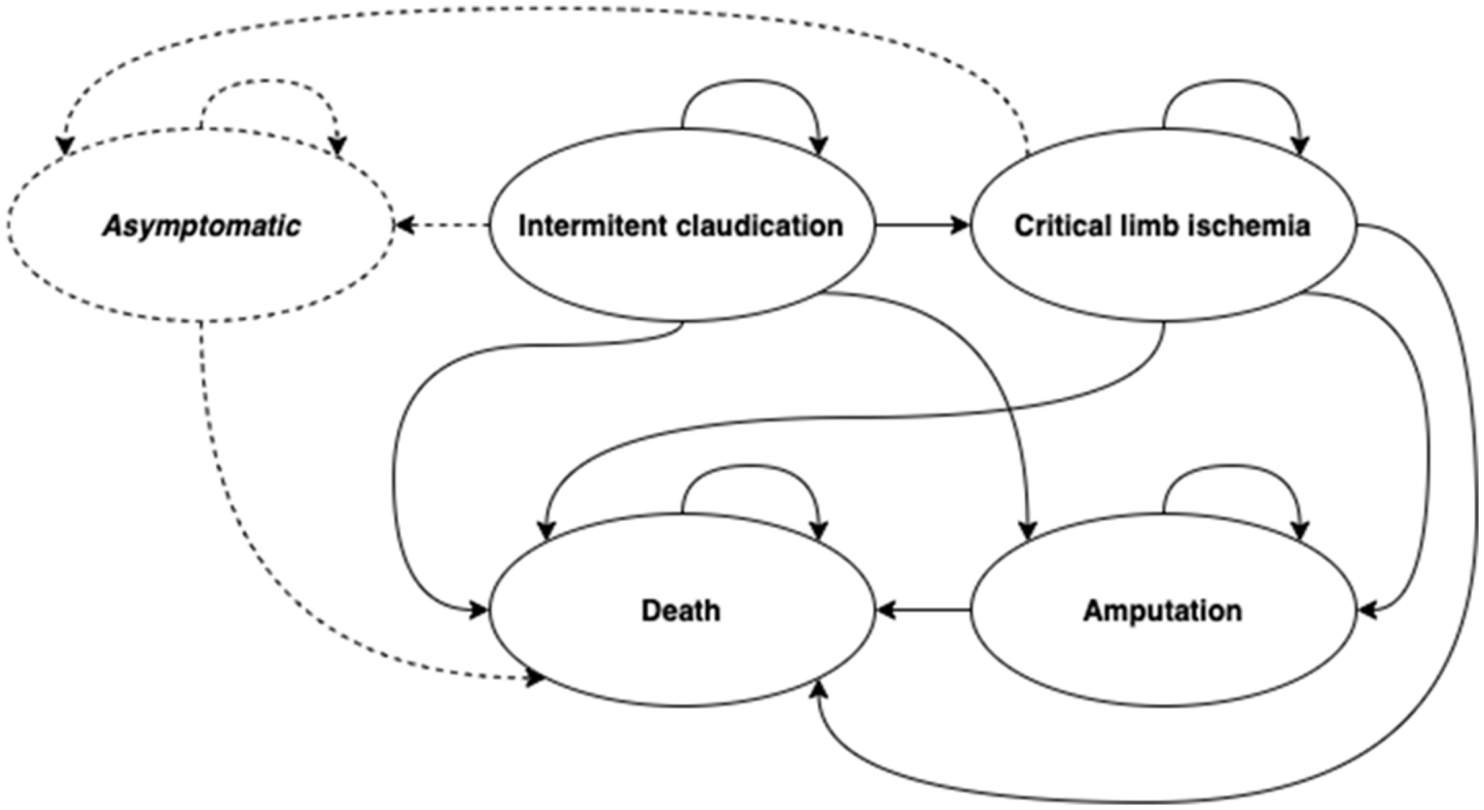
Model structure and transitions between health states.

Input data are considered for the femoro-popliteal vessels, particularly for the superficial femoral artery, if data are available. The model does not evaluate the occurrence of disease in other vessels of the limb or the occurrence of disease in the contralateral limb. The clinical states considered in the model are presented in Figure 1.

The model simulates IC, CLTI, limb amputation, patient death and, in case of successful treatment, asymptomatic condition. The sensitivity of diagnostic methods is simulated for diagnostic tests, technical success, 30-day morbidity and mortality are simulated for treatment intervention, primary patency time is simulated for successful intervention, and secondary patency time is simulated for repeated intervention. For limb amputation, the type of amputation (above/below the knee) is simulated. Different time of death of patients is simulated for different stages of the disease. The following text summarizes the structure and logic of the model and describes, in more detail, the input parameters, assumptions and the different model setting related to the evaluated diagnostic modalities. More detailed settings of other parameters are presented in Supplementary Table 1.

Compared to our former study (8), the patients did not enter the model in an asymptomatic state, and therefore they were not indicated for a screening examination. Symptomatic patients are simulated at beginning of the simulation – patients enter the model with IC or CLTI and with both limbs, but a limb may be amputated during the simulation. Since patients with lower limb symptoms are simulated, it is assumed that all patients were indicated for a diagnostic examination. In the baseline scenario, all patients are assumed to have already received pharmacological treatment. Furthermore, the model is set up in such a way that patients with the disease in the stage IIa or IIb do not undergo exercise therapy and are immediately referred for diagnosis and subsequent interventional treatment (although the first line treatment for these patients is the exercise therapy).

A cohort of 66-year-old patients was simulated with a gender distribution according to the 2021 data of the Czech Statistical Office for the simulated age group (47.2% men) (10). The cohort size was determined based on IC and CLTI prevalence data for this age group. The prevalence of IC was determined according to the information provided in the TASC II recommendation (2), and the prevalence of CLTI according to the publication by Fowkes et al. (3). A cohort of 9,546 patients (7,955 with IC and 1,591 with CLTI) was simulated from the total population of 66-year-olds of a size of 132,576 persons assuming the IC prevalence of 6% and the CLTI prevalence of 1.2% (2, 3).

To compare the diagnostic modalities, the same cohort of patients was always simulated (using the same pseudorandom numbers), where the model settings differed only in which diagnostic examination the patients were referred for. The sensitivity values of the diagnostic modalities are shown in Table 1 and were taken from published papers (6, 7). Different values for IC and CLTI were not considered in the model. In the case of a negative result of the diagnostic examination, the patient of classification III or IV [according to the Fontain classification (11)] was sent to another diagnostic examination. In the case of stage IIa or IIb, the patient should undergo a repeated diagnostic examination after a year.

**Table 1:** Sensitivity inputs for diagnostic modalities.

Diagnostic modality	Sensitivity	Sensitivity analysis	Source
DUS	86%	beta (2.03; 0.06)	(6)
DSA	100%	beta (12.13; 1.81)	(7)
CTA	96%	beta (3.04; 0.13)	(6)
MRA	96%	beta (3.04; 0.13)	(6)

DUS, duplex ultrasonography; DSA, digital subtraction angiography; CTA, computed tomography angiography; MRA, magnetic resonance angiography.

All patients with a positive diagnostic result are indicated for a therapeutic intervention. The technical success and the 30-day postoperative mortality and morbidity are simulated for each intervention. In the case of a technical success, the time of vessel patency is simulated (primary for the first successful intervention and secondary for the second successful intervention) during which the patient is in an asymptomatic state. In the case of a technical failure, the patient is indicated for a repeated intervention. A model patient can undergo a maximum of two successful interventions; in the case of two consecutive technical failures, it is assumed that the patient cannot undergo any further interventional therapy. In the case of repeated interventions, for any reason, a part of the patients are simulated unsuitable for reoperation.

The model considers the percutaneous transluminal angioplasty (PTA and PTA/S) treatment interventions and the bypass surgery. The model does not distinguish whether it is PTA with a drug-eluting balloon or with an uncoated balloon, and in the case of stent implantation, it is not distinguished what type of stent is used (plain metal stents, drug-coated stents, etc.). In the bypass surgery, a distinction is made between the treatment using an autologous vessel, and that using an artificial vascular prosthesis. In the case of an artificial vascular prosthesis, its type is not distinguished as well. Intervention outcome data were simulated separately for the populations of IC and CLTI patients, provided appropriate clinical evidence was available. If evidence was not available separately for IC and CLTI, the same values were considered for both subpopulations. The assignment to the type of intervention therapy is random in the model. The setting of parameters for simulation of transition times between states are the same as in Kamenský et al. (8) More detailed parameter settings are shown in Supplementary Table 1.

Limb amputation may occur during the simulation; the model distinguishes the below-knee and above-knee amputations (which affects the utility value and the cost of amputation). The states that the patient is going through affect the time to the occurrence of the patient’s death. To maintain consistency, the procedure proposed by Corro Ramos et al. (12) is used in the study, so that there is no increase in time to occurring of the event. Remaining life expectancy must be adjusted for the time elapsed since the simulation started and any changes in the patient’s condition. This adjustment is made by calculating the ratio of the expected life expectancy at baseline using updated values at time of event and expected life expectancy at baseline using baseline values. This ratio reflects any improvement or deterioration in the patient’s health status over time.

The costs were simulated from the perspective of a health care payer (a health insurance company). All costs were converted from CZK to EUR at the average exchange rate for Q2 2022 (24.64 CZK per EUR 1). Only direct medical costs were considered. In the baseline scenario, costs were discounted at the rate of 3%. The effect of other discount rates (0 and 5%) was analysed in the sensitivity analysis (see below). Table 2 summarizes the costs associated with the studied diagnostic modalities (incl. Their sources). All other costs considered in the model are listed in Supplementary Table S2.

**Table 2:** Cost input data for modeling diagnostic modalities.

Diagnostic modality	Costs [EUR]	Sensitivity analysis	Cost of contrast agents [EUR]	Source
DUS	47	Uniform distribution with parameter variation ±20%	–	(32)
DSA	416		6	(32, 33)
CTA	64		36	(32, 33)
MRA	259		35	(32, 33)

DUS, duplex ultrasonography; DSA, digital subtraction angiography; CTA, computed tomography angiography; MRA, magnetic resonance angiography.

Since the model, in the basic scenario, only simulates disability of one limb and one segment based on the data on the duration of the examination taken from the studies by Di Minno et al. (13) and Pollak et al. (7) (5–15 min for CTA, 20–30 min for MRA, 30 min for DSA, 30–45 min for DUS), the costs are calculated for one medical procedure (even if it is possible to repeat the medical procedure in a single day). CTA, MRA and DSA examinations require an application of a contrast agent. Available contrast agents, their recommended dosage according to the Summary of Product Characteristics (SPC), and their reimbursement were found in the database of the national regulator, the State Institute for Drug Control (SUKL) (14). The dosage depends on the area of use and on the patient’s body weight. In the basic scenario, a patient of 70 kg was considered (for both men and women).

The evaluated effect is QALY. The underlying utility values were taken from studies using the EQ-5D questionnaire, and are the same as in Table 3 (8).

**Table 3:** Utility values for health states (used as the model inputs).

Health state	Utility	Sensitivity analysis	Source
Asymptomatic 66–74 years	0.89	beta (10.11; 1.25)	(34)
Asymptomatic 75+ years	0.84	beta (15.16; 2.89)	(34)
IC (Fontaine class IIa)	0.63	beta (36.37; 21.36)	(35)
IC (Fontaine class IIb)	0.52	beta (47.48; 43.83)	(35)
CLTI (Fontaine class III)	0.44	beta (55.56; 70.71)	(35)
CLTI (Fontaine class IV)	0.40	beta (59.60; 89.40)	(35)
Below-knee amputation	0.61	beta (38,0.9; 24.54)	(36)
Above-knee amputation	0.40	beta (79.80; 319.20)	(36)

IC, intermittent claudication; CLTI, chronic limb-threatening ischemia.

ICER ratio is used to evaluate the cost effectiveness. This parameter is calculated assuming average lifetime costs and effects for the simulated cohort of patients. Based on the recommendations of the State Institute for Drug Control, the value of EUR 48,700 per QALY (CZK 1.2 million per QALY) is considered to be the cost-effectiveness (sometimes called willingness-to-pay) threshold (14).

When evaluating the validity of the model, face validity, internal validity, and cross validity were evaluated based on the ISPOR-SMDM recommendations (15). Face validity was confirmed through consultation with a cardiology expert. The model’s structure and assumptions were reviewed by clinicians and compared with other lower limb PAD models. Health technology assessment experts validated the study’s aim, modeling techniques, and cost analysis. A co-author, experienced with R but not involved in coding, assessed internal validity by verifying the simulation scripts and results. Cross validity was ensured by comparing the model’s structure with other lower limb PAD models. Furthermore, probabilistic sensitivity analysis, two-way deterministic sensitivity analysis and scenario analysis were performed to evaluate the robustness of the results based on ISPOR-SMDM recommendations (16). In the probabilistic sensitivity analysis, 1,000 iterations were simulated using the same pseudorandom numbers as in the baseline scenario, but the input parameters were changed based on pre-defined probability distributions [a detailed information on setting the parameters can be found in (8) and Supplementary materials], cost and effect differences are presented with 95% confidence interval (CI). In the analysis of the scenarios, the effect of the choice of the particular discount rate was evaluated; the effect of combining diagnostic DSAs with an interventional procedure; indications for pharmacological treatment after confirmation of the diagnosis using evaluated diagnostic modalities.

## 3 Results

The resulting modeled values of lifetime costs and effects for all compared interventions assuming the basic scenario are shown in Table 4. DUS, the intervention with the lowest lifetime costs, is used as the baseline for comparisons (expressed as differences). The differences between the effects of the evaluated therapies are quite small, which is probably due to the comparable median survival of patients (around 8.51 years for all diagnostic modalities).

**Table 4:** Cost-effectiveness of evaluated diagnostic modalities, results of the base case scenario.

Diagnostic modality	Costs [EUR]	Cost difference [EUR]	Effects [QALY]	Effect difference [QALY]	ICER [EUR per QALY]
DUS	10,778	–	5.542	–	–
CTA	10,804	26	5.554	0.012	2,167
MRA	11,184	406	5.554	0.012	33,833
DSA	11,460	682	5.562	0.020	34,100

DUS, duplex ultrasonography; DSA, digital subtraction angiography; CTA, computed tomography angiography; MRA, magnetic resonance angiography; QALY, quality adjusted life years; ICER, incremental cost effectiveness ratio.

The DSA examination was the most expensive diagnostic modality, and it is also associated with the highest effects in the form of QALYs. CTA and MRA were comparable in terms of effects, but the CTA examination is less expensive than MRA. CTA has the lowest ICER value (EUR 2,167 per QALY). All ICER values are below SUKL’s recommended cost-effectiveness thresholds (approx. EUR 48,700 per QALY).

If we compare the other diagnostic modalities with each other, the ICER for DSA compared to CTA is EUR 82,000 per QALY, and therefore DSA compared to CTA is not a cost-effective strategy. When comparing DSA with MRA, the ICER (EUR 34,500 per QALY) value is below the considered cost-effectiveness threshold. Since MRA and CTA bring the same effect, they can be compared only according to their price, which prioritizes CTA.

**Figure 2:**
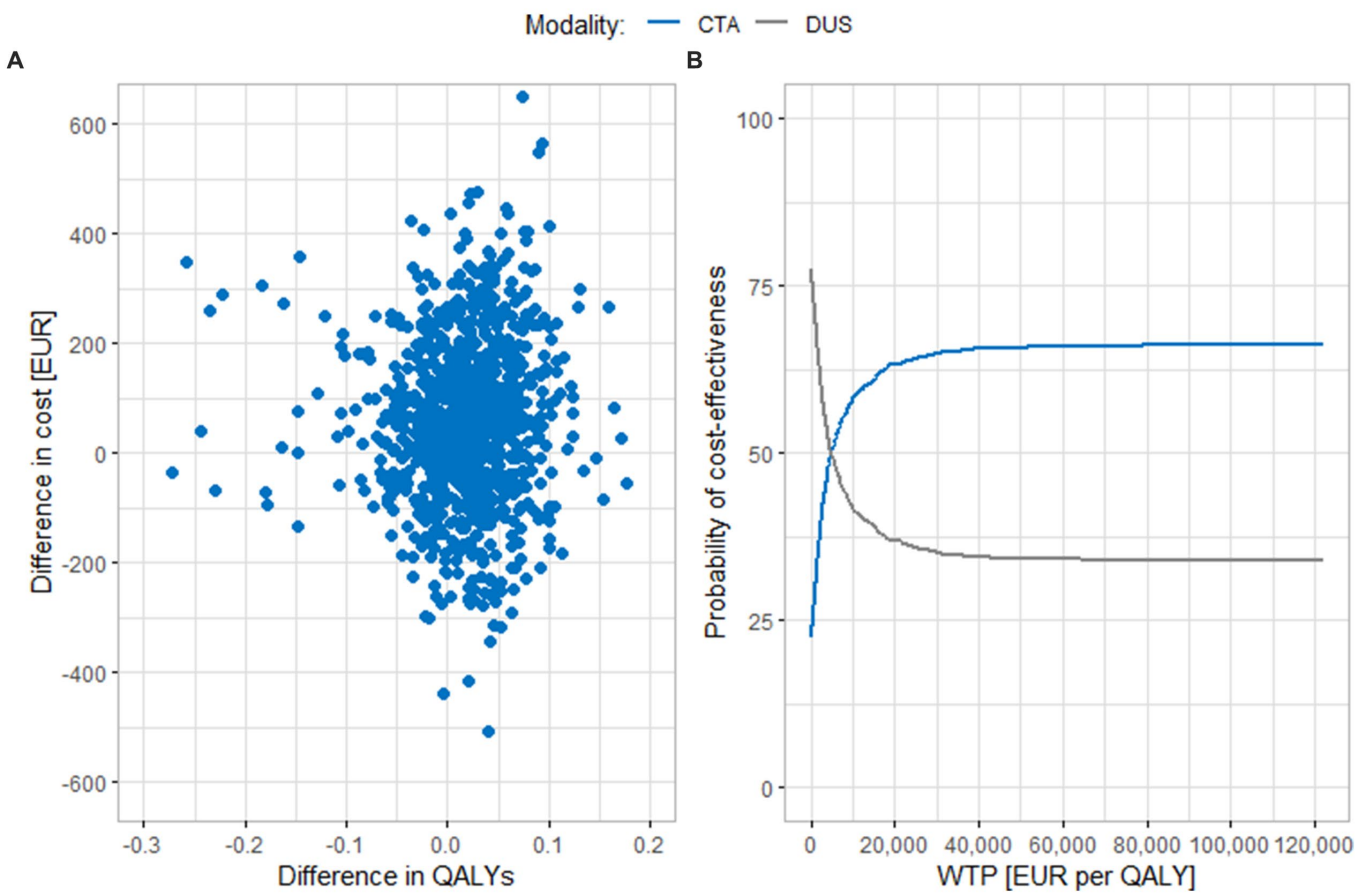
Results of the probabilistic sensitivity analysis comparing CTA and DUS. **(A)** Scatter plot of simulation results. **(B)** Cost-effectiveness acceptability curve. CTA, computed tomography angiography; DUS, duplex ultrasonography; QALY, quality adjusted life years; WTP, willingness to pay.

Figure 2 shows results of the probabilistic sensitivity analysis comparing CTA and DUS. In part A, we can see that the results of individual iterations are spread across all cost-effectiveness quadrants. Most of the iterations lie either in the upper right quadrant (the ICER values are below the cost-effectiveness threshold) or in the lower right quadrant, where CTA is more effective and less costly than DUS. The average cost difference between the compared strategies using 3% discounting is EUR 60 (95% CI EUR 50.5, 69.0). The average difference in effects is 0.0154 QALYs (95% CI 0.0116, 0.0192 QALYs), and the ICER value is EUR 3,881 per QALY. This value is close to the baseline scenario result. In part B of the figure, we can see that if the cost-effectiveness threshold was reduced to EUR 40,500 per QALY, around 65% of the results would still be cost-effective. The CTA curve does not rise further when the cost-effectiveness threshold is increased, because part of the iterations is in the upper left quadrant, where CTA is a less effective and more costly modality and DUS is the dominant intervention.

**Figure 3:**
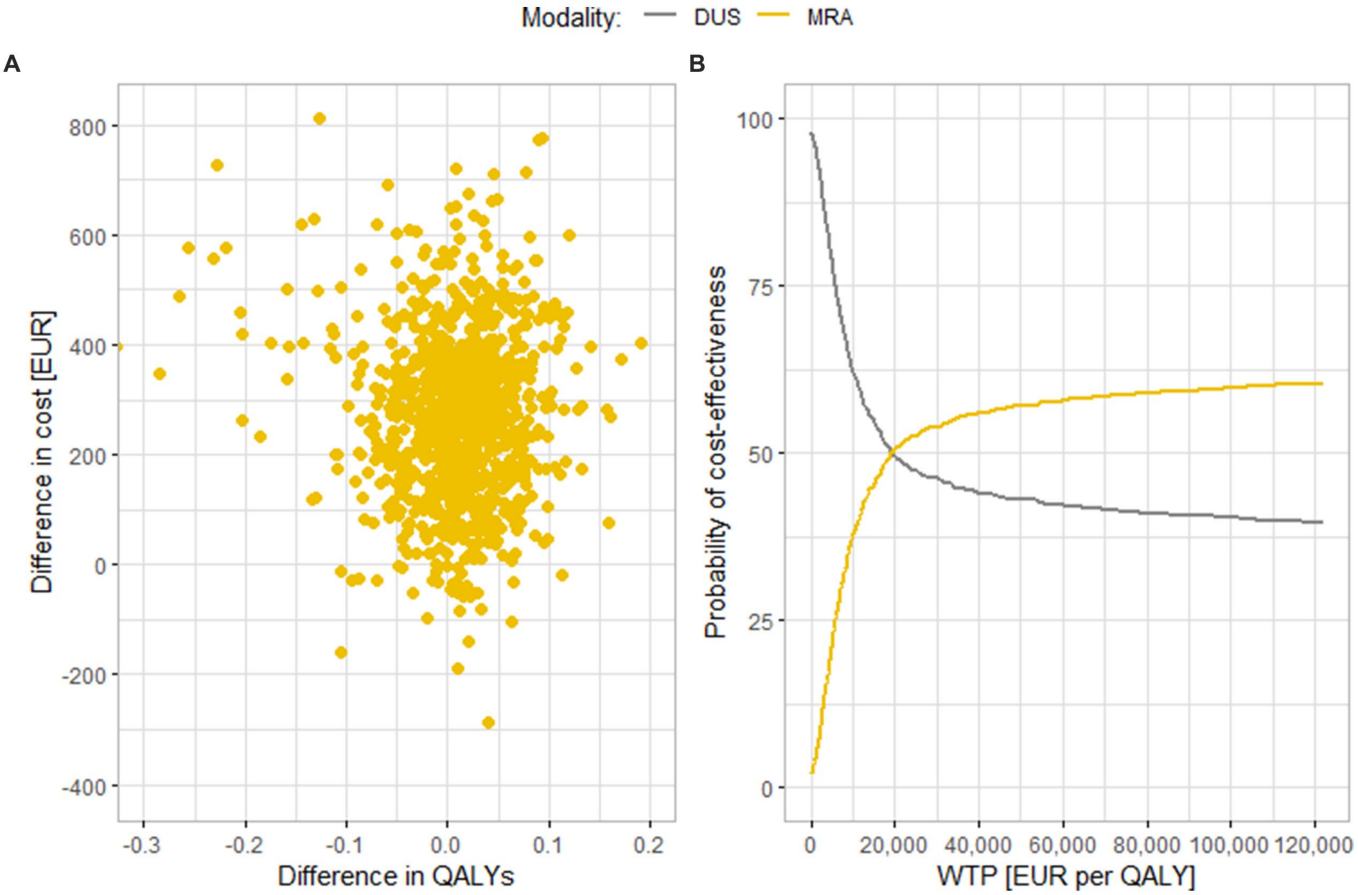
Results of the probabilistic sensitivity analysis comparing **(A)** Scatter plot of simulation results. **(B)** Cost-effectiveness acceptability curve. MRA and DUS. MRA, magnetic resonance angiography; DUS, duplex ultrasonography; QALY, quality adjusted life years; WTP, willingness to pay.

Figure 3 shows results of a similar comparison of MRA and DUS. In this case, a large part of the results turn out to be in the upper right quadrant, where MRA is a more expensive, but also more effective diagnostic modality, and also in the upper left quadrant, where MRA is a more expensive and less effective strategy. This fact is also evident in part B of the figure; if the cost-effectiveness threshold is increased up to EUR 122,000 per QALY, only 63% of iterations are cost-effective. The average cost difference is EUR 270 (95% CI EUR 260.7, 279.9), and the average effect difference is 0.0078 QALY (95% CI 0.0042, 0.0113 QALYs). The value of the ICER is thus EUR 34,904 per QALY, which is below the cost-effectiveness threshold.

**Figure 4:**
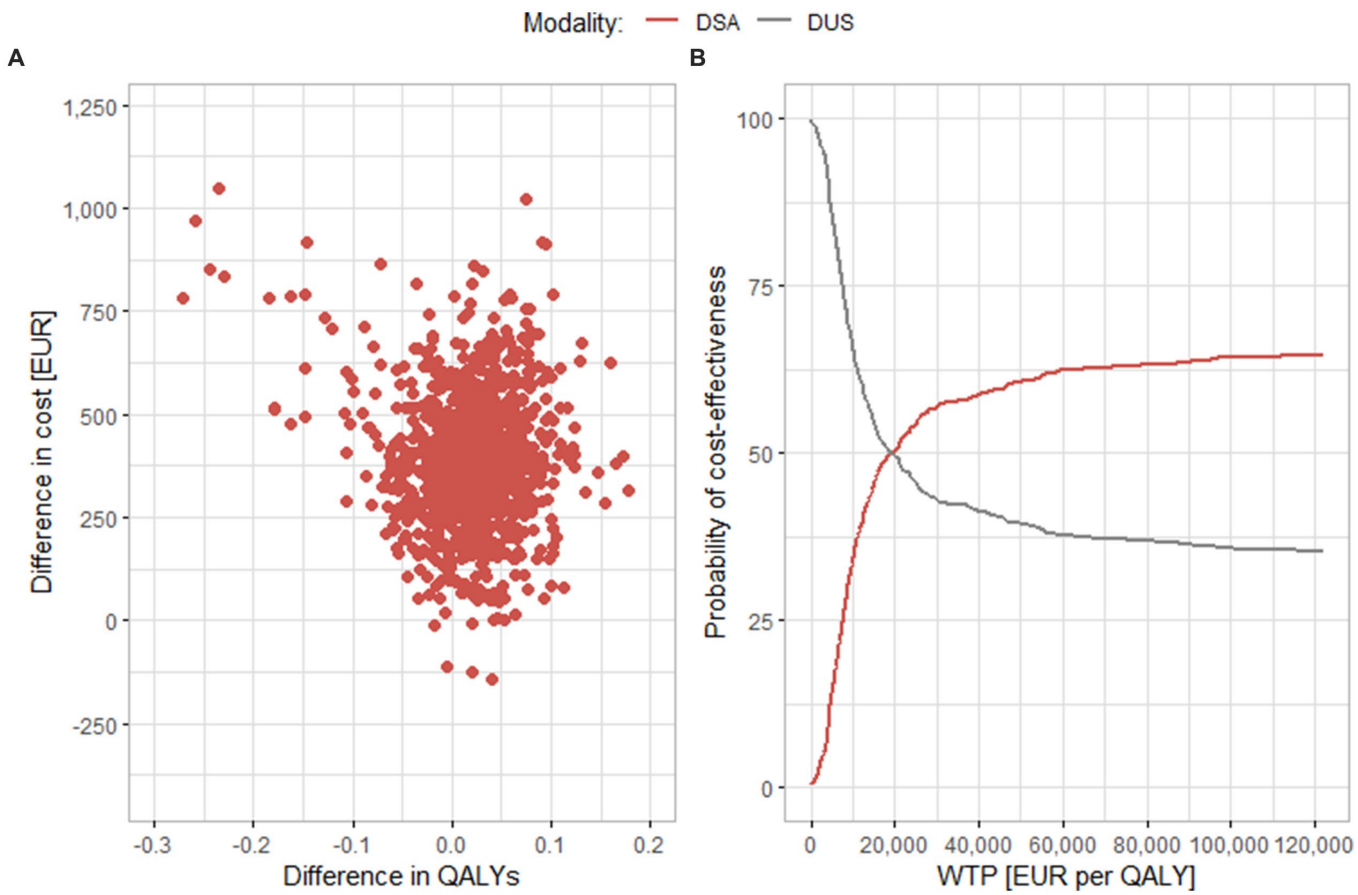
Results of the probabilistic sensitivity analysis comparing DSA and DUS. **(A)** Scatter plot of simulation results. **(B)** Cost-effectiveness acceptability curve. DSA, digital substraction angiography; DUS, duplex ultrasonography; QALY, quality adjusted life years; WTP, willingness to pay.

Figure 4 shows results of a comparison between DSA and DUS. Similar to MRA, most results are in the upper right or upper left quadrants. These findings also correspond to the results of the base scenario, MRA and DSA have similar ICER values. In part B of the figure, we can see that, like in the case of MRA, even if the WTP threshold increases, the curve does not rise further, and only around 65% of the results are cost-effective at the level of EUR 122,000 per QALY. In the sensitivity analysis, the average difference both in costs and in effects is lower than in the base scenario [EUR 392 (95% CI EUR 380.1, 404.2) and 0.0154 QALY (95% CI 0.0116, 0.0192 QALYs), respectively]. The value of the ICER is thus EUR 25,462 per QALY, which is also a lower value than in the base scenario.

When simulating a scenario where a part of the patients are treated with endovascular treatment as part of the DSA diagnostic examination, the total average cost per patient for this type of diagnosis decreases. This is because the cost of DSA is included in the cost of endovascular treatment for these patients. If 50% of patients were treated with PTA or PTA/S right at the time of diagnosis, the DSA strategy would become the least costly and remain the most effective therapy as in the baseline scenario. In this case, the DSA is the dominant strategy over all other diagnostic modalities. If at least 25% of patients underwent a combined diagnostic and therapeutic procedure, the average costs would reach the level of average costs of MRA (EUR 11,165 vs. 11,184). The ICER value, when compared to DUS, would fall to EUR 19,350 per QALY, and compared to CTA to EUR 45,125, which is below the cost-effectiveness threshold of EUR 48,700 per QALY.

Considering alternative discount rates of 0 and 5% for sensitivity testing, the order of diagnostic modalities by costs remains the same. At the 0% discount rate, CTA is again a cost-effective diagnostic modality thanks to the ICER value of EUR 2,353 per QALY, while MRA and DSA can no longer be considered cost-effective (ICER values being EUR 61,618 per QALY and EUR 49,510 per QALY, respectively). In contrast, for MRA and DSA in particular, the 5% discount rate reduced the ICER values, and as in the baseline scenario with the 3% discount rate, the values are below the cost-effectiveness threshold. The scenario with indication of the pharmacotherapy only after a diagnostic examination with positive result had the effect of increasing the ICER values for all evaluated diagnostic modalities, but all ICER values stayed below the cost-effectiveness threshold. The increase in the ICER values is mainly due to the change in costs, where the largest decrease is for DUS, because this modality has the least sensitivity. The model values for individual scenarios are shown in Table 5.

**Table 5:** Results of scenario analysis.

Diagnostic modality	Costs [EUR]	Cost difference [EUR]	Effects [QALY]	Effect difference [QALY]	ICER [EUR per QALY]
0% discount rate scenario					
DUS	11,889	–	6.467	–	–
CTA	11,905	16	6.474	0.0068	2,353
MRA	12,308	419	6.474	0.0068	61,618
DSA	12,597	708	6.481	0.0143	49,510
5% discount rate scenario					
DUS	10,200	–	5.061	–	–
CTA	10,239	39	5.075	0.014	2,786
MRA	10,601	401	5.075	0.014	28,643
DSA	10,870	670	5.083	0.023	29,130
Scenario with the indication of pharmacotherapy after a successful diagnostic examination					
DUS	10,618	–	5.548	–	–
CTA	10,734	116	5.560	0.012	9,667
MRA	11,109	491	5.560	0.012	40,917
DSA	11,410	791	5.568	0.019	41,684

DUS, duplex ultrasonography; DSA, digital subtraction angiography; CTA, computed tomography angiography; MRA, magnetic resonance angiography; QALY, quality adjusted life years; ICER, incremental cost effectiveness ratio.

## 4 Discussion

At present, various recommendations for selecting diagnostic modalities in symptomatic patients with atherosclerotic disease of the lower limb arteries are not uniform. In many cases, the choice between modalities is determined primarily by the physician’s preference or the local availability of the technology (17). The ability to predict whether (and how much) a patient will benefit from the treatment can support the physician’s decision on individual treatment. Improvements may occur through clinical outcomes or, for example, through reduction of unnecessary exposure to X-ray radiation or other adverse effects. In addition to monitoring clinical and patient outcomes, it is also necessary to evaluate the economic part of diagnostic technologies.

The use of modeling techniques appears to be a suitable approach for a comprehensive evaluation. Based on an analysis of published studies, the models appear to be often specifically aimed at solving particular research questions. When a model has been applied to a larger range of research problems, the published model has usually been adapted to address multiple research questions (18). In our study, a discrete event simulation (DES) model created to be used in solving several problems was applied to capture lifetime benefits and costs. The model was created based on the guidelines published by the societies ESC/ESVC and AHA/ACC and information from professional literature (4, 6). Our objective was to create a sufficiently complex model so that a broad spectrum of research questions can be solved, and so that there is no need to create individual (often incompatible) models for individual research questions.

The structure of the model and its assumptions were validated in collaboration with physicians from the Department of Cardiology and Angiology of the General University Hospital in Prague. The structure of the model was compared with the structure of published models focussing on diagnosis of lower limb PAD (19–23). The scripts created using the R programming language were checked as a part of the internal validity assessment by a co-author of the study. The model has already been used by the same team to evaluate the effectiveness of screening using the ABI–measurements in asymptomatic patients (8). The results of that study were compared with the results published by Vaidya et al. (24), Itoga et al. (25), and Lindholt and Søgaard (26).

Published cost analyses of diagnostic modalities in some cases focus on immediate or short-term outcomes and provide information on the costs of a correct diagnosis. However, long-term effects of a correct timely diagnosis are just as important. Hence, for diagnostic technologies, it is appropriate to analyse their effect on the entire therapeutic process, morbidity, mortality, other clinical outcomes, and (long-term) costs (18, 27, 28). For these reasons, the study evaluated cost-effectiveness of diagnostic modalities in symptomatic patients before intervention.

In the basic scenario, all evaluated diagnostic modalities (CTA, MRA, DSA) were cost-effective if compared with the cheapest option, i.e., the DUS examination with the lifetime costs of EUR 10,778. The ICER values of the analysed diagnostic modalities were below the cost-effectiveness threshold recommended by the Czech national authority [EUR 48,700 per QALY (14)]. The most expensive, but at the same time the most effective diagnostic modality is the angiographic examination (DSA). The CT examination has the lowest ICER value (EUR 2,167 per QALY), while ICER values of MRA and DSA are comparable (EUR 33,838 per QALY and EUR 34,100 per QALY, resp.). Comparing CTA with the more expensive modalities MRA and DSA found that DSA is not cost-effective if compared to CTA (EUR 82,000 per QALY), and MRA has similar results if compared to DUS. According to the results of the basic scenario, we can say that the examination using CTA seems to be the best option, as it is associated with a good efficiency at acceptable costs, and its ICER value is at a low level.

Robustness of the results was assessed using the probabilistic sensitivity analysis. In most cases, the resulting values, when simulating 1,000 iterations, were in all cost-effectiveness quadrants. Compared to DUS, the diagnostic modalities CTA and DSA performed better than MRA, where we could consider approximately 60–65% of iterations cost-effective for both modalities (they were either in the upper-right or lower-right quadrants of the cost-effectiveness plane). However, this level was still reached in CTA under the cost-effectiveness threshold of EUR 40,500 per QALY, whereas in DSA it was reached only around a high threshold of EUR 61,000 per QALY.

Last but not least, it is important to mention that if the diagnostic examination using DSA is performed together with the subsequent endovascular intervention, it becomes a cost-effective strategy. Even with the combination of diagnostic examination and endovascular intervention in 25% of patients, the value of DSA compared with CTA falls below the cost-effectiveness threshold.

A literary review found several studies evaluating cost-effectiveness of diagnostic methods for lower limb PAD using modeling techniques. Some modeling studies focused on subpopulations, typically patients with diabetes mellitus, however, they show a great heterogeneity in settings and parameters considered, which limits their applicability and comparability. None of the models used DES modeling technique to evaluate cost-effectiveness of diagnostic modalities. Simpson et al. (29) applied it to evaluate cost-effectiveness of therapeutic interventions.

Some cost comparisons were published. Yin et al. (19) compared MRA with DSA, where the diagnosis using MRA was a more costly strategy, which contradicts the conclusions of our work. This fact may be mainly due to the date of the study (1995), or the reason can be a shorter time horizon of the evaluation (2 years). Visser et al. published several studies (20–22) focused on an assessment of diagnostic modalities. Similarly to our study, they evaluated lifetime cost-effectiveness of MRA, DSA and DUS (20). In contrast to our study, however, they analysed costs from the societal perspective. As in our study, the DSA modality proved to be the most effective, and DUS the least effective. Unlike our study, MRA was less expensive than DUS, which may be due to the different perspective and different financing of health services. Another study by Visser et al. (21) is more difficult to compare with the results of our study, because the authors combined a diagnostic modality with a possible method of treatment. However, the strategy with DSA (combined with PTA, PTA/S or exercise therapy) is again associated with the greatest effect.

Collins et al. (23) compared MRA, DUS and CTA. The authors found that MRA is the cheapest strategy in a short-term perspective, but DUS in a long-term (in this case 1 year) perspective. The conclusions from the long-term evaluation are thus in agreement with our study, DUS being determined the least expensive diagnostic modality. They published similar conclusions regarding costs in their study comparing DUS and DSA (and their combinations). As well as our work, they identified DSA to be a more costly but also the most effective strategy.

It is important to mention that the presented results are valid for the femoropopliteal segment. The conclusions may differ in other segments of the lower limb. However, modifying the model to solve questions for other areas of the lower limb is not difficult. This would consist mainly in an adjustment of values simulating the effectiveness of diagnostic modalities and therapies. In the case of diagnostic modalities, this would be an adjustment of the sensitivity of the individual compared modalities.

An extension of the model can be a simulation of the occurrence of other cardiovascular events. The higher mortality of patients with PAD is captured in the model, if lower survival values have been simulated in patients with the disease (depending on the degree of disability), but the model does not simulate occurrences of such events as myocardial infarctions or cerebrovascular accidents, provided the patient has survived and is treated. If diagnostic interventions are being compared, this assumption does not have a significant effect on cost-effectiveness results. The occurrence of cardiovascular events is generally not included in simulation models for cost-effectiveness assessment (19–23, 29–31). This is because symptomatic patients are mostly simulated in these models, who are assumed to be already treated to reduce risk factors, and the imaging diagnostic methods affect the decision on the type of treatment, not the occurrence of such events.

This may cause a small bias, but the effect is usually more significant in simulations of screening cost-effectiveness. With a positive screening result, it is assumed that the patient will also be treated to prevent the occurrence of further cardiovascular events such as myocardial infarction. The cost-effectiveness results of the ABI screening examination could be even more favorable because patients with early diagnosed lower limb PAD will have a lower incidence of these events, as they are treated with, e.g., pharmacological treatment.

Another limitation may be the use of randomized controlled trials for setting model parameters. The effectiveness of the considered diagnostic modalities may be lower in real practice. This limitation is partly suppressed thanks to the DES modeling technique, where individual results from diagnostic modalities are simulated for each patient, and also thanks to the sensitivity analysis, which provides us with information about the robustness of results and conclusions.

A limitation of using the DES modeling technique and creating a model that captures the disease in the widest possible scope is that the simulations are more computer–intensive. The simulation of larger populations can take several minutes, and when simulating a large population in a probabilistic sensitivity analysis with many iterations, we can need up to hours of simulation time. This problem can be constrained by parallel computing methods.

The proposed model can also be used for the needs of early-stage HTA. Similarly to the study by Visser et al. (21), it is possible to determine minimum values of sensitivity and costs of a new diagnostic modality in order it was cost-effective. Because the same methodological basis will be used that has already proven itself in the evaluation of existing diagnostic modalities, we can assume that relevant information for an eventual implementation of the new technology can be obtained before the design and development phase of the device is completed.

## 5 Conclusion

Since the model simulates the lifelong evolution of the disease from the manifestations of intermittent claudication to the death of the patient, it is possible to carry out long-term evaluations, and thus capture all possible impacts of the evaluated technologies. When comparing diagnostic modalities, DUS examination was determined as the modality with the lowest costs and DSA modality with the highest costs. In terms of QALY, however, DUS generated the least effects and DSA the greatest effects. From the cost-effectiveness point of view, the CTA examination appears to be the optimum strategy. The results of the cost-effectiveness assessment of diagnostic modalities are associated with a large degree of uncertainty, which was analysed using the probabilistic sensitivity analysis. The resulting cost-effectiveness values depend mainly on the considered sensitivity values of the diagnostic modalities.

## Data Availability

The original contributions presented in the study are included in the article/Supplementary material, further inquiries can be directed to the corresponding author.
