# Supplementary material for: Cost-effectiveness of diagnostic imaging modalities in symptomatic patients with lower limb peripheral arterial disease: discrete event simulation model
Source: Front Public Health. 2024 Sep 3;12:1367447. doi: 10.3389/fpubh.2024.1367447 (PMC11405222; doi:10.3389/fpubh.2024.1367447)
Supplement: Supplementary file 1 [file Table_1.DOCX]

Supplementary Material

# Input data

**Supplementary Table 1:** Parameters for modeling of occurrence of simulated events

| **Parameter** | **Probability distribution** | **Values** | **Sensitivity analysis** | **Source** |
| --- | --- | --- | --- | --- |
| ***Event occurence*** | | | | |
| Occurrence of CLI (IC to CLTI) | Exponential | rate = 0.064 | Exponential parameter  varied by ± 20% with uniform distribution | (1) |
| Occurrence of amputation (Fontaine IIa, IIb) | Weibull | shape=0.40, scale=2010 | Variation of the scale parameter with uniform distribution within ± 20% interval | (2) |
| Occurrence of amputation (Fontaine III, IV) | Weibull | shape=0.46, scale=73.44 |  | (2) |
| Fontaine class distribution in occurrence of IC | NA | IIa: 66,8%; IIb: 33,2% | IIa: beta (32.53, 16.17) | (3) |
| Fontaine class distribution in occurrence of CLTI | NA | III 66,7%; IV 33,3% | III: beta (32.63, 16.29) | (3) |
| ***Death of the patient*** | | | | |
| Time to death (female) | Weibull | shape=3.52, scale=31.98 | Variation of the scale parameter with uniform distribution within ± 20% interval | (4) |
| Time to death (male) | Weibull | shape=2.64, scale=36.84 |  | (4) |
| Death after amputation | Weibull | shape=0.61, scale=5.48 |  | (5) |
| Time to death with IC | NA | RR = 3,1 relative to death | Fixed | (6) |
| Time to death with CLTI | NA | RR = 2,0 relative to IC | Fixed | (7) |
| ***Vessel patency*** | | | | |
| Primary PTA (IC) | Weibull | shape = 0.87, scale= 28.13 | Variation of the scale parameter with uniform distribution within ± 20% interval | (8–11) |
| Primary PTA (CLTI) | Weibull | shape = 0.93, scale = 10.17 |  |  |
| Primary PTA/S (IC) | Weibull | shape = 0.80, scale = 28.19 |  |  |
| Primary PTA/S (CLTI) | Weibull | shape = 0.88, scale = 9.37 |  |  |
| Primary (autologous; IC) | Weibull | shape = 1.04, scale = 31.37 |  |  |
| Primary (autologous; CLTI) | Weibull | shape = 1.16, scale = 11.66 |  |  |
| Primary (graft; IC) | Weibull | shape = 1.22, scale = 20.78 |  |  |
| Primary (graft; CLTI) | Weibull | shape = 1.42, scale = 9.01 |  |  |
| Secondary PTA (IC) | Weibull | shape = 0.79, scale = 67.10 |  |  |
| Secondary PTA (CLTI) | Weibull | shape = 0.82, scale = 21.74 |  |  |
| Secondary PTA/S (IC) | Weibull | shape = 0.83, scale = 47.21 |  |  |
| Secondary PTA/S (CLTI) | Weibull | shape = 0.87, scale = 16.09 |  |  |
| Secondary (autologous; IC) | Weibull | shape = 0.96, scale = 56.49 |  |  |
| Secondary (autologous; CLTI) | Weibull | shape = 1.01, scale = 21.96 |  |  |
| Secondary (graft; IC) | Weibull | shape = 0.97, scale = 33.00 |  |  |
| Secondary (graft; CLTI) | Weibull | shape = 1.21, scale = 14.38 |  |  |
| ***Treatment, intervention parameters*** | | | | |
| Effect of pharmacological treatment | NA | HR = 0,75 | fixed | (12,13) |
| Vein for bypass | NA | 60% autologous | beta (39.4, 26.27) | (14) |
| 30-day morbidity PTA | NA | IC = 3,8%; CLI = 35,8% | IC: beta (96.16, 2434);  CLI: beta (63.84, 114.49) | (10) |
| 30-day morbidity PTA/S | NA | IC = 2,5%; CLI = 35,8% | IC: beta (97.48, 3802)  CLI: beta (63.84, 114.49) | (15)  (10) |
| 30-day morbidity bypass | NA | IC = 9,5%; CLI = 46,3% | IC: beta (90.41, 861)  CLI: beta (53.24, 61.75) | (10) |
| 30-day mortality PTA | NA | IC = 0,2%; CLI = 2,8% | IC: beta (99.80, 49799)  CLI: beta (97.17, 3373) | (16)  (17) |
| 30-day mortality PTA/S | NA | IC = 0,4%; CLI = 2,8% | IC: beta (99.60, 24799)  CLI: beta (97.17, 3373) | (15)  (17) |
| 30-day mortality bypass | NA | IC = 0,7%; CLI = 3,3% | IC: beta (99.29, 14085)  CLI: beta (96.67, 2833) | (15)  (17) |
| 30-day mortality amputation | NA | BKA = 8,9%; AKA = 27,7% | BKA: beta (91.01, 932)  AKA: beta (72.02, 188) | (5) |
| Technical success PTA | NA | 86.5% | beta (15.16, 2.89) | (18) |
| Technical success PTA/S | NA | 95% | beta (10.38, 1.69) | (19) |
| Technical success Bypass | NA | 100% | Fixed | (20) |
| Reoperation possible | NA | IC = 95%; CLI = 98,45% | IC: beta (4.05, 0.21)  CLI: beta (0.57, 0.009) | (16,21) |
| Distribution of complication for bypass according DRG | NA | mild: 43%; severe: 57% | severe=2-3: beta (56.57, 74.99) | (22) |
| ***Amputation parameters*** | | | | |
| Amputation in CHSC | NA | yes = 46,32% | beta (53.22, 61.67) | (22) |
| Type of amputation | NA | BKA = 68,3%; AKA = 31,7% | BKA: beta (31.02, 14.40) | (16) |

IC – intermittent claudication; CLTI – critical limb threatening ischemia; PTA – percutaneous transluminar angioplasty; PTA/S – PTA with stent imlantation; DRG – diagnosis related group; CHSC – Center for highly specialized care; BKA – below-knee amputation; AKA – above-knee amputation; RR – relative risk, HR – hazard ratio.

**Supplementary Table 2:** Cost inputs for model

| Costs | Values [EUR] | Sensitivity analysis | Source |
| --- | --- | --- | --- |
| PTA | 3,116 | log-normal^1^ (8.04, 0.21) | (22) |
| PTA/S | 4,482 | log-normal^1^ (8.42, 0.24) | (22) |
| PTA, PTA/S with complication | 7,126 | log-normal^1^ (8.87, 0.20) | (22) |
| Bypass | 5,628 | log-normal^1^ (8.63, 0.06) | (22) |
| Bypass with complication | 8,092 | log-normal^1^ (8.99, 0.07) | (22) |
| Bypass with severe complication | 13,100 | log-normal^1^ (9.48, 0.10) | (22) |
| Amputation | 4,421 | log-normal^1^ (8.39, 0.04) | (22) |
| Amputation with complication | 7,047 | log-normal^1^ (8.86, 0.04) | (22) |
| Amputation in CHSC | 6,344 | log-normal^1^ (8.75, 0.05) | (22) |
| Amputation with complication in CHSC | 10,885 | log-normal^1^ (9.29, 0.05) | (22) |
| Post amputation care | 2,718 | log-normal^1^ (7.90, 0.10) | (23) |
| Prosthetic care AKA amputation | 2,535 | log-normal^1^ (7.83, 0.10) | (23) |
| Prosthetic care BKA amputation | 2,413 | log-normal^1^ (7.78, 0.10) | (24) |
| Prosthetic care-service | 635 | log-normal^1^ (6.45, 0.10) | (23) |
| Treatment ulceration and gangrene | 1,892 | log-normal^1^ (7.54, 0.10) | (25) |
| Pharmacological care | 298 | log-normal^1^ (5.69, 0.10) | (26,27) |
| Comprehensive angiologist examination | 35 | Variation of the point value; uniform distribution within ± 20% interval^2^. | (28) |
| Targeted angiologist examination | 18 |  | (28) |
| Control angiologist examination | 9 |  | (28) |
| Comprehensive vascular surgeon examination | 19 |  | (28) |
| Targeted vascular surgeon examination | 13 |  | (28) |
| Control vascular surgeon examination | 6 |  | (28) |

^1^For log-normal distribution in sensitivity parameters are presented logmean and logsd; ^2^parameters minimum and maximum; PTA – percutaneous transluminar angioplasty; PTA/S – PTA with stent imlantation; CHSC – Center for highly specialized care; BKA – below-knee amputation; AKA – above-knee amputation

# Supplementary references

1. Ezeofor V ‘Segun, Bray N, Bryning L, Hashmi F, Hoel H, Parker D, Edwards RT. Economic model to examine the cost-effectiveness of FlowOx home therapy compared to standard care in patients with peripheral artery disease. *PLoS ONE* (2021) 16:e0244851. doi: 10.1371/journal.pone.0244851

2. Moussa Pacha H, Mallipeddi VP, Afzal N, Moon S, Kaggal VC, Kalra M, Oderich GS, Wennberg PW, Rooke TW, Scott CG, et al. Association of Ankle-Brachial Indices With Limb Revascularization or Amputation in Patients With Peripheral Artery Disease. *JAMA Netw Open* (2018) 1:e185547. doi: 10.1001/jamanetworkopen.2018.5547

3. Vaidya A, Kleinegris M-C, Severens JL, Ramaekers BL, ten Cate-Hoek AJ, ten Cate H, Joore MA. Comparison of EQ-5D and SF-36 in untreated patients with symptoms of intermittent claudication. *Journal of Comparative Effectiveness Research* (2018) 7:535–548. doi: 10.2217/cer-2017-0029

4. Úmrtnostní tabulky za ČR, regiony soudržnosti a kraje - 2019–2020. *Úmrtnostní tabulky za ČR, regiony soudržnosti a kraje - 2019–2020* https://www.czso.cz/csu/czso/umrtnostni-tabulky-za-cr-regiony-soudrznosti-a-kraje-i09aftm7w4 [Accessed February 14, 2022]

5. Abry L, Weiss S, Makaloski V, Haynes AG, Schmidli J, Wyss TR. Peripheral Artery Disease Leading to Major Amputation: Trends in Revascularization and Mortality Over 18 Years. *Annals of Vascular Surgery* (2022) 78:295–301. doi: 10.1016/j.avsg.2021.04.037

6. Criqui MH, Langer RD, Fronek A, Feigelson HS, Klauber MR, McCann TJ, Browner D. Mortality over a Period of 10 Years in Patients with Peripheral Arterial Disease. *N Engl J Med* (1992) 326:381–386. doi: 10.1056/NEJM199202063260605

7. Norgren L, Hiatt WR, Dormandy JA, Nehler MR, Harris KA, Fowkes FGR. Inter-Society Consensus for the Management of Peripheral Arterial Disease (TASC II). *Journal of Vascular Surgery* (2007) 45:S5–S67. doi: 10.1016/j.jvs.2006.12.037

8. Vossen RJ, Vahl AC, Leijdekkers VJ, Montauban van Swijndregt AD, Balm R. Long-Term Clinical Outcomes of Percutaneous Transluminal Angioplasty with Optional Stenting in Patients with Superficial Femoral Artery Disease: A Retrospective, Observational Analysis. *European Journal of Vascular and Endovascular Surgery* (2018) 56:690–698. doi: 10.1016/j.ejvs.2018.06.063

9. Nishibe T, Yamamoto K, Seike Y, Ogino H, Nishibe M, Koizumi J, Dardik A. Endovascular Therapy for Femoropopliteal Artery Disease and Association of Risk Factors With Primary Patency: The Implication of Critical Limb Ischemia and TASC II C/D Disease. *Vasc Endovascular Surg* (2015) 49:236–241. doi: 10.1177/1538574415614406

10. Antoniou GA, Georgiadis GS, Antoniou SA, Makar RR, Smout JD, Torella F. Bypass surgery for chronic lower limb ischaemia. *Cochrane Database of Systematic Reviews* (2017) doi: 10.1002/14651858.CD002000.pub3

11. Ambler GK, Twine CP. Graft type for femoro-popliteal bypass surgery. *Cochrane Database of Systematic Reviews* (2018) 2018: doi: 10.1002/14651858.CD001487.pub3

12. Golledge J, Drovandi A. Evidence-Based Recommendations for Medical Management of Peripheral Artery Disease. *JAT* (2021) 28:573–583. doi: 10.5551/jat.62778

13. Bevan GH, White Solaru KT. Evidence-Based Medical Management of Peripheral Artery Disease. *Arteriosclerosis, Thrombosis, and Vascular Biology* (2020) 40:541–553. doi: 10.1161/ATVBAHA.119.312142

14. Chew DKW, Owens CD, Belkin M, Donaldson MC, Whittemore AD, Mannick JA, Conte MS. Bypass in the absence of ipsilateral greater saphenous vein: Safety and superiority of the contralateral greater saphenous vein. *Journal of Vascular Surgery* (2002) 35:1085–1092. doi: 10.1067/mva.2002.124628

15. Liang P, Li C, O’Donnell TFX, Lo RC, Soden PA, Swerdlow NJ, Schermerhorn ML. In-hospital versus postdischarge major adverse events within 30 days following lower extremity revascularization. *Journal of Vascular Surgery* (2019) 69:482–489. doi: 10.1016/j.jvs.2018.06.207

16. Simpson EL, Kearns B, Stevenson MD, Cantrell AJ, Littlewood C, Michaels JA. Enhancements to angioplasty for peripheral arterial occlusive disease: systematic review, cost-effectiveness assessment and expected value of information analysis. *Health Technology Assessment* (2014) 18: doi: 10.3310/hta18100

17. Darling JD, McCallum JC, Soden PA, Korepta L, Guzman RJ, Wyers MC, Hamdan AD, Schermerhorn ML. Results for primary bypass versus primary angioplasty/stent for lower extremity chronic limb-threatening ischemia. *Journal of Vascular Surgery* (2017) 66:466–475. doi: 10.1016/j.jvs.2017.01.024

18. Schulte K-L, Hardung D, Tiefenbacher C, Weiss T, Hoffmann U, Amendt K, Tepe G, Heuser L, Treszl A, Lau H-J, et al. Real-world outcomes of endovascular treatment in a non-selected population with peripheral artery disease – prospective study with 2-year follow-up. *Vasa* (2019) 48:433–441. doi: 10.1024/0301-1526/a000798

19. Saxon RR, Dake MD, Volgelzang RL, Katzen BT, Becker GJ. Randomized, Multicenter Study Comparing Expanded Polytetrafluoroethylene–covered Endoprosthesis Placement with Percutaneous Transluminal Angioplasty in the Treatment of Superficial Femoral Artery Occlusive Disease. *Journal of Vascular and Interventional Radiology* (2008) 19:823–832. doi: 10.1016/j.jvir.2008.02.008

20. van der Zaag ES, Legemate DA, Prins MH, Reekers JA, Jacobs MJ. Angioplasty or Bypass for Superficial Femoral Artery Disease? A Randomised Controlled Trial. *European Journal of Vascular and Endovascular Surgery* (2004) 28:132–137. doi: 10.1016/j.ejvs.2004.04.003

21. de Vries SO, Visser K, de Vries JA, Wong JB, Donaldson MC, Hunink MGM. Intermittent Claudication: Cost-effectiveness of Revascularization versus Exercise Therapy. *Radiology* (2002) 222:25–36. doi: 10.1148/radiol.2221001743

22. Stažení distribučního balíčku CZ-DRG v4 revize 1. *Ministerstvo zdravotnictví* (2021) https://www.mzcr.cz/stazeni-distribucniho-balicku-cz-drg-v4-revize-1/ [Accessed May 29, 2022]

23. Klasnová N. Ekonomická náročnost protetické a terapeutické péče dolní končetiny po transfemurální amputaci. [Diplomová práce]. Kladno: ČVUT, Fakulta biomedicínského inženýrství v Kladně. (2019).

24. Půlpán R. Úvodní slovo. *Ortopedická protetika, Odborný časopis Federace ortopedických protetiků technických oborů* (2018)

25. Stryja J, Turoň J. Wound Healing – Cost-eff ectiveness Data from the Providers and Payers Viewpoint. *Cesk Slov Neurol N* (2017) 80/113: doi: 10.14735/amcsnn2017S18

26. Ústav zdravotnických informací a statistiky. Zdravotnická ročenka České republiky 2018. (2018)

27. Wilkins E, Wilson L, Wickramasinghe K, Bhatnagar P, Leal J, Luengo-Fernandez R, Burns R, Rayner M, Townsend N. European Cardiovascular Disease Statistics 2017. Brusel: European Heart Network. (2017).

28. info@aion.cz AC-. 428/2020 Sb. Vyhláška o stanovení hodnot bodu, výše úhrad hrazených služeb a regulačních omezení pro rok 2021. *Zákony pro lidi* https://www.zakonyprolidi.cz/cs/2020-428 [Accessed May 29, 2022]
